# Oral chronic GVHD after allogeneic stem cell transplantation without total body irradiation performed at a young age

**DOI:** 10.1007/s00520-022-06836-7

**Published:** 2022-01-24

**Authors:** Kristine Eidal Tanem, Petter Wilberg, Phoi Phoi Diep, Ellen Ruud, Anne B. Skaare, Lorentz Brinch, Bente Brokstad Herlofson

**Affiliations:** 1 Department of Oral Surgery and Oral Medicine, Faculty of Dentistry, University of Oslo, Postbox 1109 Blindern, N-0317 Oslo, Norway; 2 Department of Pediatric Oncology and Hematology, Oslo University Hospital, Oslo, Norway; 3 Institute of Clinical Medicine, University of Oslo, Oslo, Norway; 4 Department of Pediatric Dentistry and Behavioral Science, Faculty of Dentistry, University of Oslo, Oslo, Norway; 5 Department of Hematology, Oslo University Hospital, Oslo, Norway; 6 Unit of Oral and Maxillofacial Surgery, Division for Head, Neck, and Reconstructive Surgery, Department of Otorhinolaryngology, Oslo University Hospital, Oslo, Norway

**Keywords:** Hematopoietic stem cell transplantation, Allogeneic, Oral complications, Oral GVHD, Long-term survivors

## Abstract

**Purpose:**

Long-term survivors (LTSs) of allogeneic hematopoietic stem cell transplantation (allo-HCT) may experience oral long-term effects like chronic graft-versus-host disease (oral cGVHD). The aim of this study was to investigate oral cGVHD in patients treated at a young age (< 30 years) more than 5 years after allo-HCT without total body irradiation (TBI).

**Methods:**

All 94 participants went through a semi-structured interview, and an oral examination. Diagnosis of oral cGVHD was based on the “National Institutes of Health (NIH) cGVHD diagnosis and staging consensus criteria” from 2014.

**Results:**

Mean age at transplantation was 17.5 years (range 0.4–29.9 years), and mean time since transplantation was 16.7 years (range 6–26 years). Oral cGVHD was diagnosed in 26 (28%) of 94 LTSs. Of which 20 (21.5%) showed lichen planus-like (LPL) changes, and additionally six (6.5%) also fulfilled the diagnostic criteria of oral cGVHD since they had one or more distinctive signs and symptoms of oral cGVHD combined with definite cGVHD in another organ. No LTSs reported oral cGVHD (NIH) grade 3. There was a significant association between cGVHD in the oral cavity and cGVHD in another organ (77% vs 29%, *p* < 0.001). Out of 72 LTSs, who answered the questions regarding taste disturbances, 16 (22%) reported dysgeusia. No LTSs developed secondary malignancies in the oral cavity during follow-up time.

**Conclusion:**

Oral long-term effects, such as oral cGVHD, may persist for many years after allo-HCT without TBI-conditioning in patients treated at a young age.

## Introduction

Allogeneic hematopoietic cell transplantation (allo-HCT) is a potentially curative treatment for both malignant and non-malignant hematopoietic diseases [1]. Conditioning regimen is an essential part of the transplantation treatment. In Norway, the conditioning regimen used has mainly been based upon myeloablative chemotherapy without total body irradiation (TBI). This is in contrast to other traditional protocols that include TBI [1].

Patients receiving allo-HCT at a younger age have better survival compared to patients treated above 40 years [2]. Even though the survival rate has increased since the first allo-HCT in the 1950s [1], long-term survivors (LTSs) may experience severe late morbidity after transplantation [3]. The most significant late effect is chronic graft-versus-host disease (cGVHD) [4]. It is an inflammatory condition, where donor lymphocytes respond to the patient’s antigens resulting in immunological reactions [5] affecting mainly the skin, oral mucosa, liver, gastrointestinal tract, and eyes [6]. GVHD may be considered acute or chronic based on clinical signs and symptoms rather than time since transplant [4]. Chronic GVHD includes two subcategories: (1) classic cGVHD, where there are no signs and symptoms of acute graft-versus-host disease (aGVHD), and (2) overlap condition, where there are both signs and symptoms of aGVHD and cGVHD [4]. The incidence of cGVHD depends on factors such as age, sex, diagnosis, donor type, human leukocyte antigens (HLA) disparity, history of aGVHD, and type of conditioning regimen [7–11].

Evidence regarding oral involvement in LTSs diagnosed with cGVHD is conflicting [7, 8, 11]. In a recent study, as many as 83% of the patients with cGVHD showed oral involvement after allo-HCT [12]. The diagnosis of cGVHD was based on the NIH criteria from 2014 (NIH 2014). These criteria consider lichen planus-like (LPL) changes in the oral mucosa as the diagnostic criterion for oral cGVHD [4]. Furthermore, LTSs may experience distinctive signs and symptoms of oral cGVHD such as xerostomia, mucoceles, mucosal atrophy, ulcers, and pseudomembranes [4]. LTSs may experience taste changes or alterations (dysgeusia) which may further reduce their quality of life [13, 14]. There is also an increased risk of developing secondary malignancies, including cancer in the oral cavity, in LTSs after allo-HCT [15].

Oral late effects after allo-HCT, and especially oral cGVHD, may cause severe discomfort and pain, which may result in problems with eating, drinking, and talking [7]. It is, therefore, important that the medical team have knowledge on risk factors, diagnostic criteria, treatment, and prognosis to correctly assess and manage the patients with oral involvement [16]. However, there is a gap in the literature regarding the nature of oral cGVHD in LTSs, especially in children and young adults, related to treatment regime [17]. In most of the published studies on oral cGVHD in LTSs, TBI is included in the treatment regime [12, 18–20]. In Norway, TBI is seldom used, and this provides an opportunity to investigate oral late effects in non-irradiated LTSs.

Thus, the aim of the present study was to investigate the prevalence, symptoms, and characteristics of oral cGVHD in patients treated at a young age (< 30 years at transplantation) more than 5 years after allo-HCT without TBI.

## Methods

### Study design and population

**Fig. 1:**
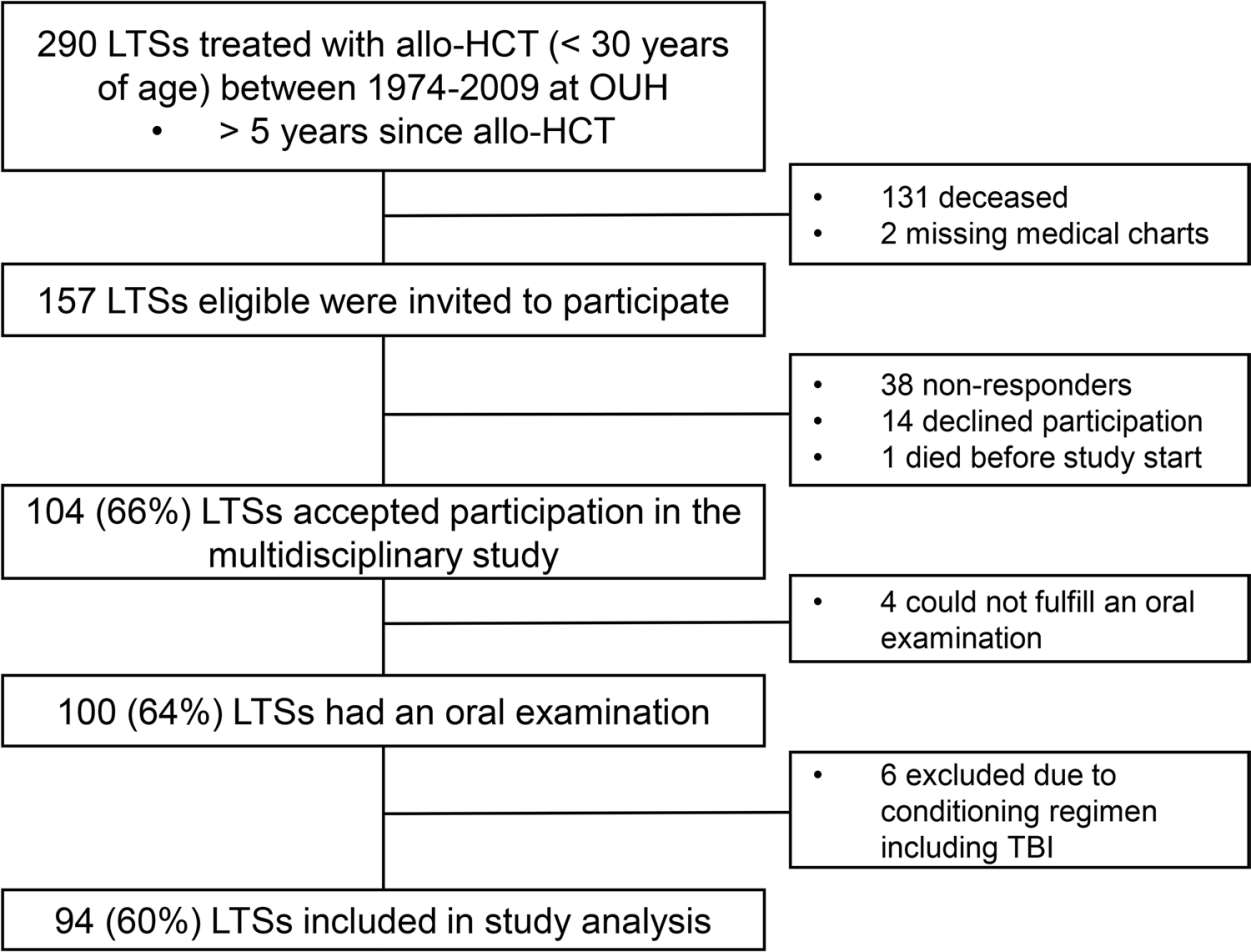
Flowchart of included study population. Abbreviations: LTSs long-term survivors, allo-HCT allogeneic hematopoietic stem cell transplantation, OUH Oslo University Hospital

Our cross-sectional, non-comparative clinical study was part of a large, national multidisciplinary Norwegian Allo Survivorship study (AlloSS-young), investigating health impairments in young survivors after allo-HCT [21, 22]. The study included subjects with leukemia, lymphoma, benign hematological diseases, immunodeficiencies, or metabolic diseases treated with allo-HCT at Oslo University Hospital, during the period April 1974 to August 2009 [21, 22]. Eligibility criteria for inclusion were (1) all patients alive and older than 16 years at study start, August 1, 2014; (2) all patients were aged < 30 years at transplantation; (3) minimum time since allo-HCT was 5 years. Exclusion criteria included diagnosis of mucopolysaccharidosis type 1 (Mb Hurler) since they may have multi-organ pathology that could influence the outcomes of interest [23]. A total of 157 LTSs met the eligibility criteria and were invited to participate in the multidisciplinary study. In total 104 (66%) out of 157 accepted to participate. Four LTSs were not able to complete the oral examination protocol, and 6 LTSs were excluded due to conditioning regimen including TBI. Hence, 94 non-irradiated subjects were included in our analyses of oral late effects (Fig. 1).

### Interview and medical history

All participants went through a semi-structured interview conducted by a dentist between August 2014 and January 2016. Xerostomia, the subjective feeling of dry mouth [24], was registered as a dichotomous variable (yes/no) after asking the participant “Does your mouth often feel dry?” Taste disturbances (dysgeusia) were registered dichotomously by the questions: “Does food and beverages taste different after treatment?” and/or “Do you experience alterations in the sense of taste?” Answering “yes” to one or both questions was regarded as dysgeusia. Only those treated with allo-HCT at an age above 10 years were asked about taste disturbances. Medical history regarding underlying diagnosis, conditioning regimen, donor, use of medications, oral secondary malignancies, and history of aGVHD was collected from each LTS’s medical history chart.

### Oral examination

All 94 subjects included in the AlloSS-young study went through a thorough oral examination performed by a dentist. This included a systematic registration of oral mucosal findings, including signs of oral cGVHD and candida infection. Clinical photographs of the oral mucosa were taken of all LTSs to document clinical oral findings.

Diagnosis of oral cGVHD was based on the “NIH cGVHD diagnosis and staging consensus criteria” from 2014 [4]. These criteria may also be applied to pediatric patients (< 18 years) [25]. The presence of oral LPL changes alone was sufficient for the diagnosis of oral cGVHD. Location of changes in the oral cavity, including vermilion lip, was registered. In addition, subjects without oral LPL changes but with one or more distinctive signs or symptoms, xerostomia, mucoceles, mucosal atrophy, ulcers, and pseudomembranes, in combination with a definite diagnosis of cGVHD in another organ, were also regarded as oral cGVHD cases [4]. Information regarding cGVHD in another organ was based on multidisciplinary examinations performed during the study [21, 22]. A 4-point (0–3) clinical scoring system was used to grade the severity of oral cGVHD; 0 = no symptoms, 1 = mild symptoms with no limiting oral intake, 2 = moderate symptoms with partial limitation of oral intake, 3 = severe symptoms with major limitation of oral intake [4].

Dry mouth was assessed by use of the mirror friction test [26], which is a screening method for saliva lubrication effect. The back of a dental mouth mirror is dragged along the buccal mucosa, and the presence of friction or no friction was registered [26].

**Fig. 2:**
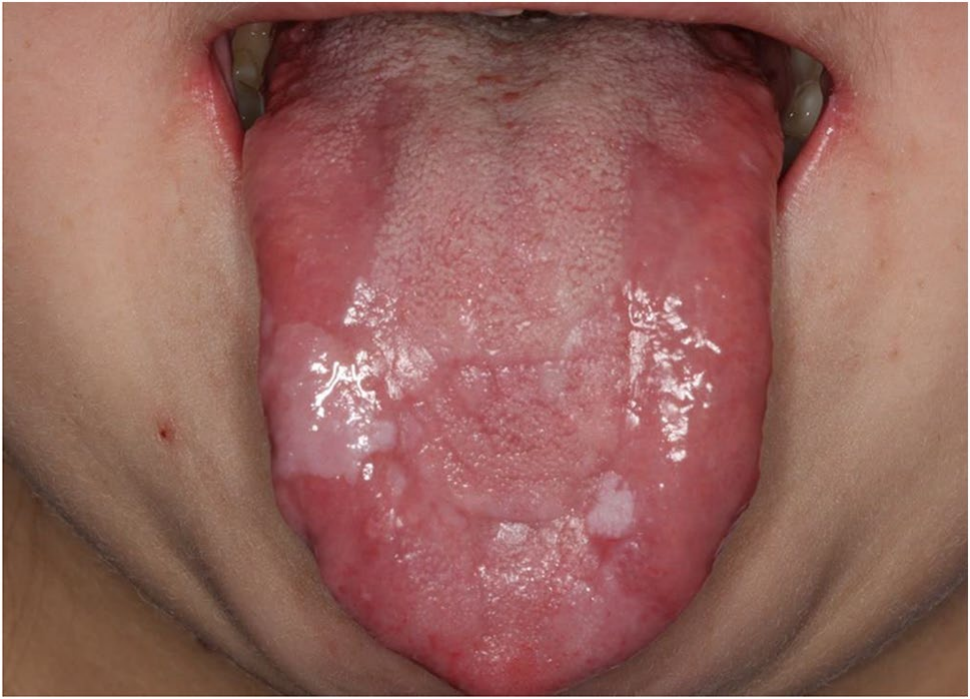
Tongue with oral cGVHD in a 19-year-old female treated with allogeneic hematopoietic stem cell transplantation at the age of 11 years. The patient provided permission for this photo

**Fig. 3:**
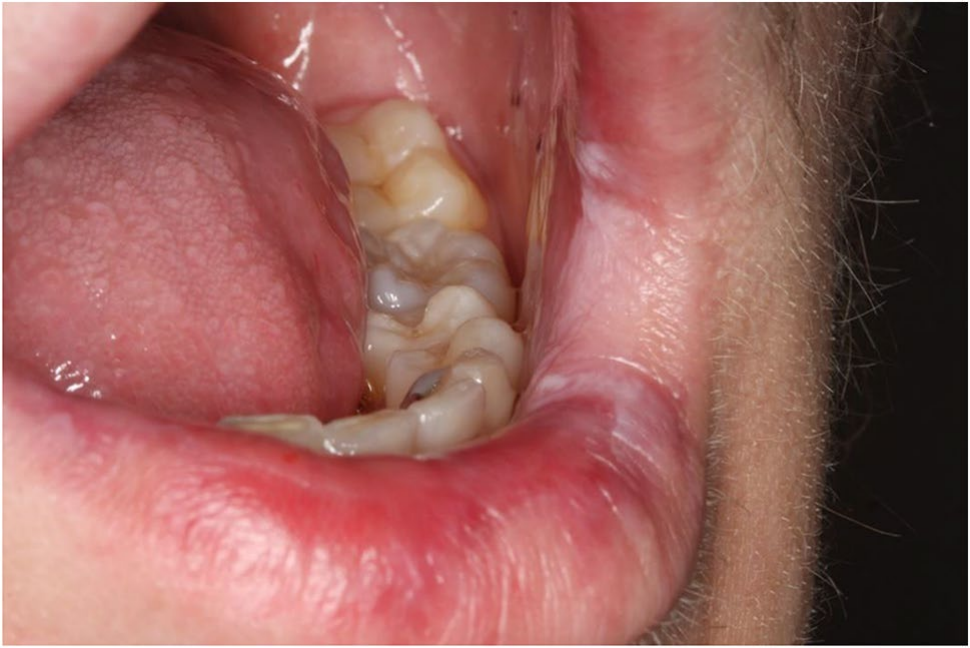
Vermilion of the lower lip with oral cGVHD in an 18-year-old male treated with allogeneic hematopoietic stem cell transplantation at the age of 13 years. The patient provided permission for this photo

Fungal carriage was identified by rubbing a sterile cotton swab over two oral mucosal sites: the left buccal mucosa and the anterior part of the tongue (Figs. 2 and 3). Samples were inoculated on CHROMagar (CHROMagar^TM^Candida, Paris/France) culture plates, incubated at 37 °C for 48 h, and a growth of ≥ 10 colonies was regarded as a positive microbiology test for fungal carriage [27]. A diagnosis of oral candidiasis and the need for antimycotic therapy were based on both positive clinical and microbiological findings.

### Statistical analysis

Descriptive statistics were used for patient characteristics and presented as mean with standard deviation (SD) and range for continuous variables, and frequencies with proportion for categorical variables. Comparison of means was performed by independent *t*-test, while comparison of proportions was performed by chi-square test or Fisher’s exact test, as appropriate. A difference was considered statistically significant when *p* < 0.05. Statistical analyses were done using IBM SPSS Statistics 24.0 for Windows (IBM Corp., Armonk, NY).

## Results

### Patient sample characteristics

The LTSs characteristics are summarized in Table 1. At study start, the mean age was 34.1 years (range 17–54 years). Mean age at transplantation was 17.5 years (range 0.4–29.9 years), and mean follow-up since treatment was 16.7 years (range 6–26 years). Most of the participants, 68 (72%) LTSs, had been treated with allo-HCT for a malignant disease. The majority had their conditioning regimen based on busulfan and cyclophosphamide (busulfan 4–5 mg/kg/day per os for 4 days and cyclophosphamide 60 mg/kg/day for 2 days (Bu/Cy2)) without in vivo T-cell depletion (Table 1).

**Table 1:** Characteristics of study population (*n* = 94)

		Oral cGvHD		
		Yes	No	*P*-value
Patients, *n (%)*	94	26 (28)	68 (72)	
Gender, *n (%)*				
Male	43 (46)	13 (50)	30 (44)	
Female	51 (54)	13 (50)	38 (56)	0.61
Age at allo-HCT, *mean* ± *SD (years)*	17.5 ± 9.4 (range 0.4–29.9)	17.6 ± 8.6 (range 0.4–29.9)	17.4 ± 9.8 (range 0.7–29.9)	0.94
< 18 years, *n (%)*	44 (47)	16 (61.5)	28 (41)	0.08
≥ 18 to ≤ 30 years, *n (%)*	50 (53)	10 (38.5)	40 (58)	
Age at study start, *mean* ± *SD (years)*	34.1 ± 11.2 (range 17–54)	33 ± 11.4 (range 18–54)	34.6 ± 11.2 (range 17–53)	0.55
Follow-up after allo-HCT, *mean* ± *SD (years)*	16.7 ± 5 (range 6–26)	15.4 ± 5.5 (range 7–24.5)	17.1 ± 4.8 (range 6–26)	0.14
Diagnosis, n (%) *				
Malignant (AML, ALL, CML, MDS, NHL)	68 (72)	22 (85)	46 (68)	0.1
Non-malignant (SAA, SCID, LAD, HLH, WAS, Thalassemia)	26 (28)	4 (15)	22 (32)	
Conditioning regimen, *n* (%) **				
Chemotherapy Bu/Cy	69 (73.5)	22 (84.5)	47 (69)	
Chemotherapy and antithymocyte globulin (ATG)	23 (24.5)	3 (11.5)	20 (29.5)	0.08
No condition, *n (%)*	2 (2.2)	1 (4)	1 (1.5)	
Donor, *n (%)*				
Sibling	56 (60)	15 (57.5)	41 (60)	
Unrelated donor (MUD***)	31 (33)	9 (35)	22 (32)	0.94
Other related (haploidentical)	7 (7.4)	2 (7.5)	5 (8)	
Oral cGvHD, *n*		26	68	
Oral lichen planus-like changes (OLPLC), *n (%)*	20 (21.5)	20 (77)	na	
Distinctive signs and symptoms of oral cGvHD, *n (%)*	40 (43)	26 (100)	14 (21)	
Xerostomia	25 (26.5)	12 (46)	13 (19)	
(Use of medications with dry mouth as a side effect)	25 (26.5)	9 (34.5)	16 (23.5)	
Mucosal atrophy	16 (17)	15 (58)	1 (1.5)	
Mucoceles	5 (5.3)	2 (8)	3 (4.5)	
Oral ulcers	6 (6.4)	5 (19)	1 (1.5)	
Pseudomembranes	1 (1.1)	1 (4)	0	
History of acute GvHD, *n (%)*	47 (50)	15 (58)	32 (47)	
Oral mucosa	4 (4.3)	4 (15)	0	0.36
Chronic GvHD in other organ, *n (%)*	40 (43)	20 (77)	20 (29)	< 0.001
Other oral findings, *n (%)*				
Reduced saliva lubrication effect	11 (12)	6 (23)	5 (7)	
Candidaiasis, ≥ 10 colonies	4 (4.3)	1 (4)	3 (4)	
Dysgeusia (allo-HCT > 10 yrs), *n (%) n* = 72	16 (22) of 72	6 (26) of 23	10 (20) of 49	

^*^AML acute myeloide leukemia, *CML* chronic myeloid leukemia, *ALL* acute lymphoblastic leukemia, *MDS* myelodysplastic syndrome, *NHL* non-Hodgkin lymphoma, SAA severe aplastic anemia, *SCID* severe combined immunodeficiency, *LAD* leukocyte adhesion deficiency, *HLH* hemophagocytic lymphohistiocystosis, *WAS* Wiskott-Aldrich syndrome ** *Bu* busulfan, *Cy* cyclophosphamide,***MUD matched unrelated donor

### GVHD diagnosis

In total, 26 (28%) of all LTSs met the NIH diagnostic criteria of oral cGVHD (Table 1). Oral LPL changes were identified in 20 (21.5%) of LTSs. All the 20 LTSs who had LPL changes also had other distinctive signs and symptoms of oral cGVHD (Table 1). In addition to these 20 LTSs with LPL changes, six (6.5%) had one or more distinctive signs and symptoms of oral cGVHD in addition to definite cGVHD in another organ, five with ocular affection and one with both ocular and skin affection [21, 22]. Hence, they also fulfilled the diagnostic criteria of oral cGVHD (Tables 1, 2).

**Table 2:** Long-term survivors (LTSs) with oral cGVHD (*n* = 26)

Oral lichen planus-like changes, *n (%)*	20 (77)
Site of oral lichen planus-like changes	
Buccal mucosa	
Unilateral	3
Bilateral	7
Gingiva	
Maxilla	3
Mandibula	3
Tongue	12
Palate	5
Lips	9
Distinct signs and symptoms of oral cGVHD + cGVHD in another organ,*n (%)*	6 (23)
Ocular cGVHD	5
Ocular + skin cGVHD	1
Scoring of oral cGVHD (NIH 2014),*n (%)*	
Grade 0 = no symptoms	13 (50)
Grade 1 = mild symptoms with no limiting oral intake	9 (34.5)
Grade 2 = moderate symptoms with partial limitation of oral intake	4 (15.5)
Grade 3 = severe symptoms with major limitation of oral intake	0

### Characteristics of LTSs with oral cGVHD

Most of the LTSs with oral cGVHD, 16 (61.5%), were treated < 18 years of age, though no significant relation was found between oral cGVHD and whether they were treated < 18 years or ≥ 18 to < 30 years (Table 1). There was a significant association between cGVHD in the oral cavity and cGVHD in another organ (77% vs 29%, *p* < 0.001) (Table 1). No relations were seen between gender, age at transplantation, age at oral examination, follow-up time, diagnosis, conditioning regimen, history of aGVHD, and donor type and the occurrence of oral cGVHD (Table 1).

The most common oral locations of LPL changes were the buccal mucosa and the tongue. Using the clinical scoring system, no LTSs reported LPL changes grade 3 (Table 2). Among LTSs who scored grade 2, xerostomia was the most frequent distinctive symptom.

### Other oral signs and symptoms

Fourteen (21%) LTSs, who did not fulfill the oral cGVHD NIH diagnostic criteria, were registered with one or more distinctive signs and symptoms associated with oral cGVHD (Table 1). Among these, xerostomia was the most frequently reported symptom by 13 (19%) LTSs. Some participants used medications in which dry mouth is a possible side effect (Table 1).

Seventy-two LTSs, 23 with oral cGVHD, were older than 10 years at allo-HCT and they were all asked about taste alterations. Among those, dysgeusia was reported by 16 (22%) of 72 LTS, of which six (26%) of 23 with oral cGVHD (Table 1). A positive mirror friction test was registered in 11 (12%) of all LTSs, six of them had oral cGVHD (Table 1). Oral candidiasis, based on clinical and microbiological findings, was diagnosed in four (4.3%) out of all LTSs (Table 1). All four used medications may have a high risk of inducing candidiasis as an adverse effect (glucocorticoids). None of the LTSs included in the analysis had a history of a post-transplantation oral squamous cell carcinoma (SCC).

## Discussion

To our knowledge, this is the first study to investigate oral late effects, including oral cGVHD, after allo-HCT without TBI in young recipients (all patients < 30 years at transplantation) using the 2014 NIH criteria at a mean follow-up of 16.7 years post-HCT. The present study showed that 26 (28%) LTSs fulfilled the criteria of oral cGVHD [4]. Twenty LTSs had oral LPL changes, and six had distinctive signs and symptoms of oral cGVHD in addition to cGVHD diagnosed in another organ [4]. The prevalence found in this study is lower than previously reported in the literature [7, 8]. However, Dahllöf and coworkers [20] reported similar findings in which 20% of patients younger than 12 years developed oral cGVHD [20]. Oral cGVHD was reported by Treister and coworkers (2005) in 45% out of 49 pediatric patients, and by Hull and coworkers (2012) in 56% out of 88 patients aged between 19 and 65 years [18, 19]. In a recent study, using the 2014 NIH criteria, 83% out of 77 patients with cGVHD had oral cGVHD involvement [12]. In contrast to our study, all these studies also included subjects treated with TBI-conditioning, and with a shorter mean follow-up time (below 5.8 years) [12, 18–20]. These factors, in addition to differences in population sizes, diagnostic criteria, patient’s age, sex, diagnosis, treatment, donor type, human leukocyte antigens (HLA) disparity, and history of aGVHD [7–10, 19], may have an impact on the results which make comparison difficult [19].

Even though we found no significant relationship between the presence of oral cGVHD and follow-up time (*p* = 0.14), symptoms of oral cGVHD seem to become less prominent with time [28]. Especially in survivors with mild or moderate cGVHD, remission seems more likely to occur compared to survivors with severe cGVHD [28]. In our study, using the NIH scoring of cGVHD, 50% of the subjects with oral cGVHD reported no symptoms, and no one reported severe symptoms (grade 3) [4]. The most frequently affected sites of LPL lesions were the buccal mucosa and the tongue. This is in accordance with another study, where the buccal and labial mucosa and the tongue were the most frequently reported affected sites in adult LTSs after allo-HCT [29]. We found a significant relationship between oral cGVHD and cGVHD in another organ; this indicates the importance of an oral examination in those patients diagnosed with a general cGVHD.

Dysgeusia in patients undergoing allo-HCT is a known adverse effect that usually resolves within 3 months after treatment, but it may persist [30, 31]. Normative data regarding taste disturbances in the Norwegian population do not exist, but data from a German adult population (mean age 52 years) estimate taste impairment to be around 20% [32], and 17% taste impairment in an American population (≥ 40 years) [33]. In the current study, 16 (22%) of 72 LTSs (treated with allo-HCT > 10 years of age), of which six (26%) of 23 LTSs with oral cGVHD, reported persisting dysgeusia. Hull and coworkers reported reduced taste perception after allo-HCT in 20% of all participants in their study [18]. In another study, which included 91 patients after (≥ 3 months) allo-HCT, taste disorders were observed in 47% and were significantly associated with oral cGVHD [31]. Conversely, when taste was assessed up to 3 years post-transplant by Boer and coworkers, no correlation was shown between taste disturbances and the presence of oral cGVHD in allo-HCT patients [34]. Thus, it is unclear whether an association between taste disturbances and the presence of oral cGVHD exists. It should be taken into consideration that dysgeusia may be affected by hyposalivation [35], and the lack of objective quantitative measurement of salivary function in this study is an important limitation. Other factors that may influence dysgeusia are oral hygiene, mucosal infections, dental pathologies, diet, and tobacco use [35].

LTSs after allo-HCT at a young age have an increased risk of developing secondary malignancies, included cancer in the oral cavity [15]. The most frequent secondary malignancy in the oral cavity is squamous cell carcinoma (SCC) and it may account for late death in 10% [36, 37]. In the present study with non-irradiated participants, no LTSs developed oral SCC during the follow-up period. This could be due to the non-TBI-based conditioning regimen. Bhatia and coworkers found that patients who received TBI had a 25-fold increased risk of oral cancer compared to the general population [38]. In addition to TBI, known risk factors for developing secondary malignancies in LTSs are presence of cGVHD and treatment at a very young age (< 10 years) [15, 36, 39–41]. The mortality risk increases with time since transplant, and continues to increase even 20 years post-transplantation [37]. Due to the higher risk of developing a new primary cancer, it is recommended that LTSs have an annual, thorough examination of the oral cavity [15, 16, 36].

Limitations of the present study include lack of information regarding pre-transplantation baseline oral examination, wide range in time from transplantation to oral assessment, and lack of a control group. The long time since transplantation may introduce bias related to the risk of LTSs declining participation due to time since treatment, or that LTSs with the most severe acute or chronic GVHD died years prior to the onset of the study. Furthermore, there is a risk of underreported aGVHD in medical charts since aGVHD may have been misdiagnosed as conditions with similar clinical signs and symptoms, such as infections or drug reactions [42]. The diagnosis of oral cGVHD in this study was based on clinical examination only, no biopsy and histological examination were performed to confirm the oral diagnosis. Nicolatou-Galitis and coworkers indicated a high diagnostic value of oral clinical examination in pediatric cGVHD [43]. However, oral cGVHD may resemble other immunological disorders [4], and thus, a risk of misdiagnosis may be possible. Oral lichen planus (OLP) is a chronic inflammatory disease, affecting up to 2% of the general population with debut between the ages of 30 and 60 years [44]. Histopathological examination of biopsies from patients with oral cGVHD and OLP shows no significant differences in CD4-positive and CD8-positive T-cells, Langerhans cells, or CD68-positive cells [45]. Due to both the clinical and histopathological similarities, gathering the medical history of allo-HCT is important to distinguish between the two.

In conclusion, oral late effects may persist for many years after allo-HCT in patients treated at a young age. This study found that almost one-third of LTSs had oral cGVHD. However, the symptoms were subtle in most participants. This may indicate that severe adverse effects may become less bothersome with time after transplantation. Despite this, as a proportion of allo-HCT LTSs have oral complications, and the potential risk of developing oral cancer, a lifelong regular oral evaluation by dental professionals is essential.

## Data Availability

If requested, data and material from the current study can be available from the corresponding author.
